# Study of Structural and Multifunctional Characteristics in Dynamic Transesterification-Aided Epoxy Carbon Nanocomposites

**DOI:** 10.3390/polym18192318

**Published:** 2026-09-22

**Authors:** Vaishnav B, Priyanka Halsi, Ajay Gupta, Sravendra Rana, Sarathlal Koyiloth Vayalil

**Affiliations:** 1 Department of Physics, Applied Science Cluster, UPES, Dehradun 248007, India; 2 Deutsches Elektronen-Synchrotron DESY, Notkestr. 85, 22607 Hamburg, Germany

**Keywords:** vitrimeric nanocomposites, waste valorisation, ultra-small angle X-ray scattering, conductive elastomers, self-healing

## Abstract

Vitrimeric nanocomposites (VNCs) are futuristic materials exhibiting multifunctional properties with sustainable capabilities. However, achieving filler-derived functionalities harmoniously with their dynamic characteristics is highly momentous in VNCs. Herein, the vitrimeric thiol-epoxy matrix incorporating tyre-waste-derived carbon nanosheets (CNS) is shown as a versatile nanocomposite integrating flexibility, high strength, and tunable electrical conductivity, together with self-healing and shape-memory characteristics. The transesterification-aided bond exchange reactions enabled vitrimeric features, while the CNS endorsed electrical transport, mechanical, thermal, and dimensional stabilities. Primary investigations, together with ultra-small angle X-ray scattering, explored the structural integrity of dispersed CNS. The hierarchically dispersed nanofillers provided efficient conductive pathways, offering a systematically varying electrical conductivity with filler concentration. They exhibited excellent dimensional stability even after multiple healing cycles, along with an exceptional stretchability up to 276%. Compared with the pristine matrix, a threefold increase in storage modulus has been observed at the maximum filler loading (15 wt%). They exhibited a commendable balance between the rigidity offered by CNS and excellent matrix flexibility, showing their versatility. As a result of accelerated thermoresponsive bond-exchange reactions, a complete shape recovery with an impressive healing efficiency of 99.6% is observed. This study not only provides a detailed elucidation of a multifunctional system but also offers practical insights for developing highly efficient epoxy-based vitrimers suitable for a wide range of applications.

## 1. Introduction

Extensive research into next-generation vitrimeric systems has been prompted by the unrelenting need for multifunctional polymer composites with extensive capabilities. Due to their remarkable potential to combine the permanent crosslinking of traditional thermosets with the reprocessability and self-healing of thermoplastics, covalent adaptable networks (CANs) have become highly attractive [1]. Since the emergence of vitrimers in 2011, which showed a class of dynamic materials (CANs) undergoing associative bond exchanges [2], researchers have demonstrated various functional routes to produce similar materials, such as Diels-Alder’s [3,4], imine exchange [5,6], disulfide exchange [7,8,9], transesterification [10,11], etc. These bond-exchange reactions between the dynamic covalent bonds (DCBs) allow network rearrangement above the topological transition temperature while preserving structural integrity below it. This is in contrast to conventional thermosetting polymers that suffer from irreversible network degradation and environmental persistence. The remarkable potential of vitrimers creates numerous opportunities for the development of high-performance, sustainable materials that can be used in wearable electronics [12,13], aerospace and structural adhesives [14,15], soft robotics [16], with regenerative capabilities.

Despite these benefits, virgin vitrimers frequently possess low electrical conductivity and poor mechanical reinforcement, which limits their applications. Adding nanofillers has emerged as a key strategy to improve electrical and thermomechanical characteristics [8,11,17]. Through efficient stress transfer and filler–matrix interfacial interactions, carbon-based nanomaterials, such as carbon nanotubes [7,18], graphene [19], and carbon nanosheets, have shown remarkable efficacy in facilitating electrical conductance while concurrently enhancing modulus, strength, and toughness in elastomers. Recently, Zhang et al. [18] demonstrated a highly stable electrical conductance in flexible polyimine vitrimer by incorporating multi-walled carbon nanotubes; Liu et al. [20], on the other hand, have presented an approach to increase the mechanical and functional capabilities of vitrimeric ethylene–vinyl acetate copolymer by dispersing graphene oxide sheets within the matrix. Despite the advantages posed by such nanoparticles, their high production costs, energy-intensive synthesis procedures, and environmental issues related to raw materials produced from fossil fuels prevent commercial carbon nanofillers from being widely deployed. This has initiated a growing interest in sustainably produced carbon nanomaterials extracted from various waste streams [21,22].

With billions of end-of-life tyres produced each year and insufficient recycling infrastructure to handle their intricate vulcanised rubber composition, tyre waste poses a massive environmental burden. In the current picture, they can be converted into useful carbonaceous nanomaterials, accomplished through recent developments in catalytic conversion and chemical degradation techniques [23,24,25]. These tyre waste-derived carbon nanomaterials offer a circular economy pathway that transforms environmental contaminants into useful nanofillers. These nanofillers are not expected to outperform commercial carbonaceous fillers with higher aspect ratios, such as carbon nanotubes or graphene, yet they offer balanced advantages, while offering high surface area, good electrical conductivity, and improved mechanical characteristics, along with their sustainability and cost benefits. For these reasons, we prepared sheet-like carbon nanostructures via a two-stage controlled pyrolysis, a relatively straightforward pathway for producing carbon nanosheets [24]. Herein, we demonstrate that adding CNS to vitrimer matrices offers a strong dual advantage: improving multifunctional performance and addressing waste management through material valorisation.

Moreover, the reversible bond-exchange processes that allow network reconfiguration and defect closure upon external stimuli-induced activation give rise to remarkable capacities, including shape memory, self-healing, weldability, and reprocessability in vitrimeric nanocomposites (VNCs). The inclusion of nanofillers in such matrices not only enhances mechanical, thermomechanical, and transport properties but also influences their dynamic properties. In a typical study, Krishnakumar et.al. [26] showed the effect of GO introduction on the critical temperatures of epoxy/GO composites. Meanwhile, their atomistic simulation study on a similar composite system revealed an enhancement in self-healing characteristics through reduction in glass transition temperature (*T_g_*) [27].

For logical material design and property optimisation, it is crucial to comprehend the structural properties of nanofiller-dispersed vitrimeric composites at different length scales. While traditional characterisation methods like atomic force microscopy and transmission/scanning electron microscopy offer useful morphological information, they frequently lack the sensitivity to resolve subtle nanoscale dispersion heterogeneities, filler-aggregate interactions, and network-filler topological relationships that are confined well within the surfaces. Ultra-small-angle X-ray scattering (USAXS) has become a potent analytical tool that offers a statistically rigorous estimation by probing buried structural characteristics within polymer nanocomposite systems, from a few tens to a few hundreds of nanometres [28,29]. Moreover, there are very few studies on such vitrimeric composites that use USAXS as a tool for elucidating structural conformations. A few of our preceding studies have investigated various factors determining the structural conformation of different dimensional carbon nanofillers [30,31,32] within different matrices, motivating a similar approach for the current VNC system. The quality of nanofiller dispersion, aggregate size distributions, and the degree of nanoscale ordering can all be quantitatively evaluated using USAXS. Such parameters are crucial in determining the effectiveness of the electrical transport pathways, mechanical reinforcement, and dynamic network behaviour in carbon-filled VNCs. Therefore, a robust linkage between nanoscale architecture and macroscale multifunctional characteristics has been established with a deep structural characterisation using USAXS.

Epoxies are widely valued for their remarkable mechanical strength, excellent adherence to a range of substrates, outstanding resistance to heat and chemicals, and effective electrical insulation. These characteristics enable a wide range of applications in adhesives, coatings, structural composites in automotive and aerospace, electronics packaging, and building materials where performance and durability are crucial [33]. Imparting vitrimeric functionality in such a matrix would greatly improve its applicability for advanced functional applications. For that reason, the transesterification-based thiol-epoxy vitrimer has been prepared by introducing thiol-based cross-linkers that give rise to dynamic bond exchange characteristics via thermal activation. The resulting vitrimeric thiol-epoxy network retains the distinctive features of epoxies while adding dynamic functionalities, including the capacity to relax internal strains, reconfigure shapes, and enable self-healing and reprocessing at high temperatures, while maintaining network integrity.

Compared to epoxy-amine thermosets, the thiol-epoxy vitrimer design offers several advantages: the thiol-epoxy reaction proceeds rapidly under mild conditions, enabling low-temperature curing and precise control over network architecture; the high functionality of typical thiol and epoxy precursors yields densely crosslinked, robust networks with good mechanical integrity and chemical resistance, while the inherent hydroxyl/ester chemistry supports the transesterification dynamics. Furthermore, the thiol-epoxy reaction’s ‘click’ nature facilitates easy formulation, precise stoichiometric control, and excellent compatibility with fillers and nanomaterials, which is especially useful in nanocomposite systems where interfacial bonding and network homogeneity are critical to achieving desired multifunctional performance [34].

This study investigates the hierarchical structure of the resultant thiol-epoxy/CNS nanocomposites using USAXS. To our knowledge, no previous study has used USAXS to systematically explain the multiscale structural organisation of this carbon-filled thiol-epoxy vitrimer system produced from waste tyres. The findings from the USAXS analysis reveal structural features over many length scales that are difficult to obtain using standard microscopic methods. Furthermore, the impact of CNS addition on mechanical and thermomechanical properties has been thoroughly investigated. We evaluate the composites’ shape memory and self-healing efficiency, demonstrating their potential for novel applications. This discovery is unique not only in developing a waste-derived carbon-filled vitrimer but also in establishing a structure–property relationship through extensive multiscale structural characterisation.

## 2. Materials and Methods

### 2.1. Materials

The materials used for synthesis, including Bisphenol-A diglycidyl ether (BADGE) (340.41 g/mol), trimethylolpropane tris(3-mercaptopropionate) (TMPMP ≥ 95%), and Tin (II) 2-ethylhexanoate (92.5–100%), were purchased from Sigma-Aldrich, St. Louis, MO, USA. The waste tyre from which the nanofillers were derived was retrieved from a nearby disposal area. A polytetrafluoroethylene (PTFE) sheet (thickness: 1 cm), obtained from Sigma-Aldrich, was etched to the required geometry and used as a casting mould. The other chemicals and solvents used during synthesis were procured locally.

### 2.2. Preparation of Thiol-Epoxy/CNS Nanocomposites

The vitrimeric epoxy/CNS nanocomposite films were prepared by infusing carbonaceous nanofillers into the epoxy monomer, followed by thermal curing. The CNS nanofillers were prepared from the established catalytic pyrolysis method explained in the literature [35]. Typical steps involved in the synthesis are presented in the Supplementary Information. The prepared CNS powders were thoroughly dispersed in ethanol by ultrasonication at 40 kHz for an hour to de-agglomerate the nanofillers. A calculated amount of the dispersion was then mixed with the epoxy resin (BADGE) at 60 °C under continuous stirring. Here, the amounts of nanofiller introduced were 5, 8, 10, 12, and 15 weight percentages (wt%) of epoxy, denoted as ECx throughout this article, with x denoting the nanofiller concentration in the composite. The rigorous stirring continued at the same temperature until all the solvent had evaporated, leaving a homogeneous dispersion, to which a carefully optimised amount of thiol cross-linker (TMPMP—50 wt% of resin) and a tin-based catalyst (6.7 wt% of resin) were added. These additions, respectively, induce crosslinking of epoxides in BADGE at a higher reaction rate. After thorough mixing, the dispersion was cast on a polytetrafluoroethylene (PTFE) mould of dimensions 50 × 10 × 1 mm^3^, followed by degassing to remove any trapped air. The cast dispersion was subjected to pre-curing at 80 °C for 2 h, followed by stepwise curing at 120 °C for 1 h and then at 150 °C for 2 h, yielding freestanding thin films. Also, a pristine epoxy film without nanofillers was prepared (denoted as EP) to study the effects of CNS inclusion on the composite’s properties.

### 2.3. Characterization Techniques

The prepared free-standing films underwent the following analyses to elucidate structural, chemical, and interfacial characteristics. A wide-angle X-ray diffraction study was conducted to investigate the crystalline and phase characteristics of epoxy/CNS composites using a laboratory Cu Kα source on a Panalytical Empyrean diffractometer, Almelo, The Netherlands, operating in grazing-incidence geometry. The incident angle was fixed at 2.5°, with a *2θ* scan range from 10° to 60° at a step size of 0.06° and an exposure time of 3 s. The Raman spectra of the nanofillers and the composites were recorded using a LabRAM HR Evolution Raman spectrophotometer, HORIBA, France, between 300 and 3000 cm^−1^, with an excitation wavelength of 532 nm and an operating power of around 1 mW. The micro-morphological characteristics of the embedded fillers were imaged using a Zeiss Gemini 560 field-emission scanning electron microscope, Oberkochen, Germany, operating at an accelerating voltage of 30 kV. The surface of the films was carefully polished to elude the outermost layers for imaging the embedded nanofiller conformations.

The electrical conductivity of the composites was measured using a Keithley sourcemeter (model 2450, Keithley instruments, Solon, OH, USA) using the two-probe method. Ohmic contacts were established using thin copper strips adhered to the PTFE moulds at a distance of 20 mm, over which the dispersion was cast and cured. Such an electrode integration has advantages over surface-adhered contacts, including a constant working distance and prevention of delamination issues, while avoiding the need for Ag paste. Rather than measuring a single potential resistance, a voltage sweep (*V-I* curves) was performed for rigorous estimation of the conductance.

Thermomechanical characterisations were performed using the Thermomechanical Analyser (TMA)—TMA Q400 by TA Instruments, Eschborn, Germany. Rectangular specimens measuring 15 × 5 × 1 mm^3^ were measured under inert atmospheric conditions in dynamic mode using a three-point bending geometry. In this mode, the temperature-dependent storage modulus (*E*′), loss modulus (*E*″), and tan δ were obtained by applying a sinusoidal stress on the samples under a constant preload force and recording their phase lag (*φ*) and the complex modulus (E*), from which the parameters were derived using the following relationships:

$$E^{\prime }=E^{*}cos\left(\varphi \right);E^{\prime \prime }=E^{*}sin\left(\varphi \right)\;and\;tan\;\delta =\frac{E^{\prime \prime }}{E^{\prime }}$$

The measurements were performed over the temperature range of 30 °C to 100 °C, at a ramp rate of 3 °C per minute with a preload force of 0.01 N at a frequency of 1 Hz. The tensile measurements were carried out using an in-house-developed programmable stretching device, whose details can be found elsewhere [30,36]. The stress–strain measurements were performed at a gauge length of 15 mm at a strain rate of 2 mm per second.

The synchrotron-based ultra-small angle X-ray scattering (USAXS) studies were performed from the MiNaXS (Micro- and nanofocus X-ray scattering) beamline—P03 of PETRA III, DESY, Germany [37]. The USAXS measurements were conducted at an X-ray energy of 12 keV, corresponding to a wavelength of 1.033 Å, with a spot size of 29 × 21 µm^2^ at the sample. The scattered X-ray profiles were collected using a PILATUS 2M detector (Dectris, Baden, Switzerland) with a pixel size of 172 µm^2^ positioned at 9407 ± 1 mm from the sample. Such a large distance was chosen to access the lower scattering vector (*q*) values corresponding to the characteristic aggregate sizes of nanofillers present in the composite (~few hundred nanometres). The varying intensity as a function of *q* is given by the relation

$$q=\frac{4\pi }{\lambda }\sin\theta$$

Here, *λ* is the wavelength of the X-rays used, and *θ* is half the scattering angle. The recorded 2D detector images were processed using the DPDAK software suite (version 1.5.9) [38] to yield 1D scattering curves. In our transmission-scattering experiments, the intensity varies radially from the beam centre, allowing us to integrate the intensity concentrically (each ring corresponds to a constant *q*) to obtain *I* vs. *q* plots. This process is widely referred to as ‘cake integration’. The integrated curves then underwent a conventional data curation process, as mentioned elsewhere [39]. Unlike microscopy, X-ray scattering provides information through inverse-space imaging, which maps the Fourier transform of the scatterers’ electron density. Model-fitting approaches are also performed in the inverse space to yield or simulate a function of distribution (electron density in our case) to elucidate the shape, size, and distribution. Here, the structural information of scatterers was extracted from the data through an iterative model-fitting analysis performed using the SasView software (version 6.0.0) [40]. The programme offers a library of form and structure factor functions that can be seamlessly integrated to fit a wide range of scattering data from various structures.

## 3. Results and Discussions

The studies performed on the composites are broadly discussed in two parts. The initial sections discuss the fundamental structural and thermomechanical counterparts, whereas the analyses of functional properties are discussed later.

### 3.1. Physicochemical Properties of Epoxy/CNS Nanocomposites

Preliminary characterisations have been performed on the CNS, pristine thiol-epoxy matrix (EP), and their composites (ECx) to elucidate intrinsic structural and interfacial features. Firstly, to observe crystalline and phase characteristics, the WAXD study was conducted. The typical WAXD patterns of the samples are shown in Figure 1a. For CNS, a broad peak centred at a 2θ of ~24.6°, corresponding to (002) graphitic planar reflections, indicates exfoliation and partial graphitisation resulting from the pyrolytic post-reduction [41]. A broad peak centred at ~43.0° corresponds to reflections from the (100) planes, indicating a turbostratic, few-layer stacking [41]. A characteristic broad hump spanning between ~13° and 25° has been observed for the neat vitrimeric epoxy matrix corresponding to the scattering from cured, amorphous polymeric chains [42]. Composites, on the other hand, display a broad convolution of the matrix and CNS characteristics spanning between ~13° and 28°, which persists across concentrations. This has been widely attributed to the substantial exfoliation of graphitic layers induced by the intrusion of polymeric chains [43,44], which implies an affinitive filler–matrix interface.

The Raman spectra revealed the distinctive signatures of CNS nanofillers, neat EC, and their composites, highlighting disordered graphitisation and filler-matrix interactions. The spectra are shown in Figure 1b. The spectrum of CNS shows the characteristic *D* band at 1350 cm^−1^ and the *G* band at 1580 cm^−1^, providing information about defective and highly ordered sp^2^ carbon lattices. The *D* band arises from the breathing mode of zone-boundary phonons with A_1g_ symmetry, emphasising defects, vacancies, and turbostratic disorder, while the *G* band results from E_2g_ vibrations of sp^2^ domains [41]. The intensity ratio between the *D* and *G* bands (I_D_/I_G_) was found to be 0.82, together with a diminished *2D* band, indicating a significant defect density and partial restoration of the graphitic structure post-pyrolysis, consistent with conventional pyrolytic conversions [45]. From the spectrum shown in Figure 1b, the characteristic peaks of the cured EP and its composites are evident as follows. The peaks at 2875 cm^−1^ and 3065 cm^−1^ correspond to the symmetric stretching of aliphatic -CH_2_ and the breathing of the epoxide ring, respectively, and overlap with the CH stretching. Additionally, the epoxide ring vibration has been observed at 1454 cm^−1^, along with vibrations overlapping with the -C-O-C- ether stretching indicated by the peak at 1231 cm^−1^. Moreover, the peaks at 1113 cm^−1^ and 1609 cm^−1^ corroborate the presence of phenyl groups, and their out-of-plane bending is signified by the peaks at 638 cm^−1^ and 826 cm^−1^, respectively [46]. The diminished CNS features in WAXD and Raman spectra are partly associated with nanofiller dilution and overlap with the amorphous epoxy contribution, indicating modification in the graphitic ordering and CNS–polymer interactions within the composites. The pyrolytic CNS’s heterogeneous surface and comparatively disordered graphitic structure would render surface imperfections and irregularities for mechanical interlocking and matrix adsorption [47].

### 3.2. Structural Conformation of CNS Within Thiol-Epoxy Matrix

Macroscopic characteristics of the composites, including mechanical, thermal, and most strikingly, electron transport, are strongly governed by the confinement of nanofillers within the matrices. Essentially, the presence of functional moieties in thiol-epoxy, along with the turbostratically disordered graphitic nanofillers with relatively higher surface energies, leads to stronger interfacial interactions. Furthermore, the conformation of nanofillers is governed exclusively by the balance between strong interfacial interactions and the intrinsically persistent inter-filler (Van der Waals) interactions. Hence, we performed a deliberate investigation of CNS conformation within the dynamic matrix. From electron-microscopic images of carefully sectioned specimens, we observe that the fillers are distributed in a polydisperse fashion at all concentrations. Figure 2a shows the surface morphology of the as-prepared EC5 film. At relatively lower filler loading, as shown in Figure 2b,c, most of the aggregates are dispersed throughout the matrix space; meanwhile, at higher concentrations, larger agglomerates are evident (Figure 2d–f). To deepen our structural elucidation, we further performed sophisticated synchrotron-based X-ray scattering studies.

The USAXS profiles for the composites, along with the fitted curves, are shown in Figure 3a. The data obtained from the experiments were fit using a shape-independent Beaucage model [48,49]. The respective model is well known for elucidating hierarchical scatterers similar to our present system. It is also referred to as the unified model since it combines the Guinier factors in the lower *q* region—related to the size of the scatterers—and the Porod features in the higher *q* regime corresponding to the interfacial characteristics of the scatterers at multiple levels of scattering. Each level relates to a component of the structural hierarchy, which, upon elucidation, reveals the size and shape of the scatterers at each level. The model relates scattered intensity as a function of *q*, as follows.

$$I\left(q\right)=\sum_{i=1}^{N}\left[G_{i}\exp\left(-\frac{q^{2}R_{gi}^{2}}{3}\right)+B_{i}{\left(\frac{1}{q_{i}^{*}}\right)}^{P_{i}}\exp\left(-\frac{q^{2}R_{g\left(i+1\right)}^{2}}{3}\right)\right]+Background$$

where *R_gi_* and *P_i_* are the radius of gyration and Porod’s exponent of each level, with *G_i_* and *B_i_* being their respective pre-factors. The Guinier pre-factor, *G*, is related to the electron and number density of the particles in the respective domain, and the power-law pre-factor, *B*, is related to the surface-to-volume ratio of scatters along with their morphology. Note that the subscript *i* is an index to the respective level, denoted in the order of smaller to larger structural levels in the hierarchy. The term *q*_i_^*^ appearing in the power law is defined as follows.

$$q_{i}^{*}=q{\left[\mathrm{erf}\left(\frac{kqR_{gi}}{\sqrt{6}}\right)\right]}^{-3}$$

The respective error function has been introduced to ensure a smooth transition between the Guinier and Porod regimes. Here, the parameter *k* is a constant, with a value of around 1 decided by the Porod exponent. One could infer that each level possesses four parameters; hence, for a multi-level system, the fitting routine becomes highly intricate. In our case, we have performed the fitting for two levels of scatter population, executed using the DREAM algorithm of SasView.

From the fitted scattering profiles, we observe that with increasing nanofiller concentration, two distinctive structural features at 0.03 Å^−1^ and 0.005 Å^−1^ evolve. This clearly indicates the presence of a two-level structural hierarchy, which is pronounced at higher concentrations due to increased inter-filler interactions resulting in CNS aggregation. Being an inverse space scattering profile related to the real space particle sizes as D=2π/q, the high *q* feature is associated with the primary CNS morphologies, while the low *q* features correspond to the multiply aggregated primary structures. Figure 3b illustrates the extrema of the unified model, indicating the levels of hierarchy. The quantification of structural aspects is thoroughly done by iterative model fitting analysis using the Beaucage model. Similar multilevel (hierarchical) aggregations, which specifically prevail in the fractal-like carbon nanoparticle conformations, have been widely studied using the Beaucage model [28,50,51,52]. One understands that fractals are self-similar, repetitive structures that possess a similar complexity at all levels, and the unified model expediently elucidates such structures [53].

To obtain the parameters, we started with free-variable (unconstrained) batch fitting, including all the curves, where initial values for parameters that are highly consistent with observed extrema of *q* are obtained. Following this, deliberate individual fits are performed to elucidate the best-fit parameters for the composites. The unified fit for two levels yielded two *R_g_* and *p* values relating to the size and nature of particle morphology, which are listed in Table 1. The complete details of the fitting are shown in Table S1 of the Supplementary Materials.

The model fit has elucidated the presence of a few particle primary structures with a mean *R_g_* of ~23.6 nm, along with Porod exponents close to 2 at lower concentrations and approaching ~3 at higher concentrations, signifying a highly irregular surface conformation that reflects the fractal characteristics of the embedded nanofillers [48,54]. These primary structural moieties are the unagglomerated nanofillers, which are prominently segregated by the matrix from inter-filler interactions. Concurrently, secondary aggregate structures in the hierarchy, ranging from ~115.8 to ~126.8 nm, are observed, with aggregate sizes increasing with increasing CNS concentration. These aggregates exhibit a similar fractal geometry, indicated by exponents close to 3. Unlike an exponent of 4, which symbolises smooth surface fractals with sharp interfaces, a value close to 3 indicates rough interfaces, which is attributed to turbostratic epoxy/CNS interfaces resulting from exfoliation, as evidenced by our preliminary analyses. These observations align with the fractal-like surface structure often described for the intrinsically corrugated nature of graphitic carbon nanoparticles [55,56].

Altogether, this reflects hierarchical conformation of pyrolysis-derived CNS within thiol-epoxy, dominated by rough, irregular interfaces rather than well-defined geometries—a result with direct implications for stress transfer efficiency and interfacial adhesion, which evidently relates to the enhancement effects in the composites, including mechanical, thermomechanical, and certainly the transport characteristics.

### 3.3. Thermomechanical Properties

All thermomechanical analyses of composites were conducted using the three-point bending test in TMA. The dynamic oscillation mode was employed to measure the *T_g_* over the temperature range of 30 °C to 100 °C. As depicted in Figure 4b, the tan δ vs. temperature plot highlights the transition from a solid glassy to a flowy, rubbery state. For virgin EP, the *T_g_* was observed at 54 °C, which eventually dropped to 50 °C for EC15. The incorporation of CNS significantly enhanced the modulus, demonstrating their physical reinforcement through the interaction with the soft epoxy matrix. It is observed that the largest CNS concentration has resulted in a modest decrease in *T_g_* (~4 °C). A similar decoupling between modulus augmentation and *T_g_* decrease has been reported for similar systems, where carbonaceous filler-induced reinforcement can coexist with changes in interfacial free volume, morphology, or local segmental mobility [57,58]. As a result, the slight *T_g_* drop reported here could be attributed to heterogeneous interfacial regions and altered local chain dynamics rather than a reduction in the nanocomposite’s overall stiffness [58]. The storage modulus test was further measured over the same temperature range as shown in Figure 4a. It represents the ability of the composite to store elastic energy during deformation, which supports deformation resistance and allows elastic recovery. The neat EP recorded a storage modulus of 4.6 GPa, which increased with filler concentration, leading to 13.3 GPa for the maximum filler loading. This increasing trend highlights enhanced mechanical reinforcement with filler incorporation, indicating a superior interfacial interaction between the CNS and the dynamic epoxy matrix. However, we see that the sample softens rapidly for a feeble increment in temperature, which results from its *T_g_* being relatively close to room temperature (as observed from tan δ curves). Furthermore, the spatial arrangement of CNS may influence their modulus behaviour, as observed due to their embedded scaffold within the matrix.

### 3.4. Functional Characteristics of Epoxy/CNS Composites

#### 3.4.1. Self-Healing, Shape Memory Characteristics

The presence of thermally activated DCBs facilitates self-healing and shape memory characteristics, along with surface welding, in the prepared films. The catalyst in the prepared samples functions as a Lewis acid, encouraging thiol-epoxy interactions by initiating the thiol group’s nucleophilic attack on the oxirane ring. Through oxirane ring-opening reactions, this interaction ultimately results in the creation of thiol-ether bonds. Additionally, ester moieties and the hydroxy groups produced during crosslinking take part in the transesterification events [59]. Upon thermal activation, these covalent bonds facilitate dynamic transesterification, giving the material an intrinsic capacity to mend itself. A schematic representation of the explained mechanism is shown in Figure 5.

Firstly, the self-healing capability of the composites has been evaluated, as presented in Figure 6a. As a representative sample, EC15 films have been bisected, after which they have undergone complete healing by bringing the cut pieces into contact at 90 °C for 30 min. This relatively rapid healing is attributed to the efficient thermal activation, which facilitated rapid bond formation between the thiol esters and epoxy-bound alcoholic moieties. Furthermore, a quantitative analysis of multiply cut-healed samples has been performed by assessing the mechanical characteristics with healing cycles. The temperature-dependent moduli measured for the EC15 composite between 30 °C and 100 °C, for two cut-and-heal cycles (shown in Figure 6d), indicated that the moduli of the as-prepared EC15 have evolved from 13.3 GPa (for virgin EC) to 12.1 GPa and then to 10.6 GPa, recovering 91.4% and 80.0% during the first and second healing cycles, respectively. This progressive decrement is attributed to the incomplete rebonding and irreversible re-aggregation of CNS during successive healing cycles [60], similarly inferred from the conductivity analyses presented in the following section (see electrical transport studies).

As shown in Figure 6c, the surface adherence of EC15 has been studied for a very small overlapping surface of 8 × 10 mm^2^ at 90 °C at different heating times. The tensile results indicated efficient rebonding between the surfaces by recovering almost its initial tensile strength (Figure 6e). The recovery of tensile strength after heating and treating the intact surfaces for 20 min was relatively reduced due to the incomplete rebonding at the interface. Consequently, the film underwent shear slippage, which can be seen from its stress–strain curve. After a thorough healing of up to 40 min, the ultimate tensile strength and maximum elongation have marginally shifted from 32.48 MPa and 276% to 32.35 MPa and 266%, recovering 99.6% of its strength with a slight reduction in its stretchability. As observed in scattering studies, the dispersed CNS intrinsically possesses higher interfacial sites, which enhance the distribution of applied heat throughout the matrix.

After evaluating the self-healing capability, shape memory tests are conducted on the EC15 composite as shown in Figure 6b. Here, the as-prepared sample has been programmed to the desired shape through thermal activation of bond exchanges and fixed by cooling to room temperature. The shape recovery ratio (*R_r_*) of the composites was calculated using the following equation:

$$R_{r}=\;\frac{\theta_{m}\;-\;\theta_{r}}{\theta_{m}}\times 100\%$$

Here, *θ_m_* is the deformed angle to which it has been thermally programmed, and *θ_r_* is the angle of recovery upon thermal activation [61]. It has been observed that the programmed shape of the EC15 composite is highly stable and results in a complete shape recovery. From the obtained data, the EC15 composite has shown 100% recovery (calculation is included in the Supplementary Materials), signifying its dynamic efficacy. At a higher temperature of 120 °C, rapid shape recovery has been observed (shown in the Supplementary Video). As explained previously, the shape-memory effect arises from the combination of a permanently crosslinked vitrimer network and a thermally activated dynamic transesterification chemistry. Below the critical temperature, the exchangeable ester bonds are effectively “frozen”, where the epoxy vitrimer behaves as a conventional covalent network; the permanent network stores elastic strain energy during deformation, which defines the permanent shape and provides the driving force for recovery [62]. When the programmed specimen is heated, the segmental mobility increases and transesterification reactions become active, allowing the network to relax local stresses, enabling the temporary shape to revert to the memorised permanent shape under the stored elastic energy.

#### 3.4.2. Electrical Transport Studies

CNS dispersed within the thiol-epoxy matrix tends to form continuous pathways, facilitating electron transport under an applied potential. The conductivity of the prepared films was inferred from the *V-I* characteristics, shown in Figure 7a. The nanofiller concentrations used in this study fall within the critical percolation regime, where a consistent increase in conductivity with filler loading is observed. Through established percolation networks, the epoxy films evolve from a highly insulating matrix (~10^−14^–10^−12^ S.m^−1^) to a highly conductive composite in the order of 10^−6^ S.m^−1^. We observe (from Figure 7b) that the conductivity increased from ~ 2 µS.m^−1^ at 5 wt% to 28 µS.m^−1^ at 15 wt% loading, displaying a 14-fold increase between the respective concentrations. At such filler loadings, nanosheets offer dense conductive channels, where the electron hopping between neighbouring filler particles (in the initial concentrations) and the dynamic electron transfer via interconnected nanosheets (near saturation) are facilitated [63]. From the obtained values, we infer that the percolative networks are not saturated, residing in the system’s operational regime, facilitating tunability in electrical properties.

Through nanosheet reorientation during thermal processing and the preservation of conductive pathways by self-healing of microcracks, the vitrimeric network’s dynamic covalent bond exchange allows the regeneration of broken network pathways in disrupted composites. To examine the respective behaviour, the conductance of a median EC8 sample has been measured after a cut-heal cycle (as shown in Figure 7c). It is observed that the complete bisection disrupted the overall conductance, which recovered to ~31% of its initial value upon thermal healing. It can be seen that the system shows an inclined mechanical recovery, yet a partial recovery of net electrical conductance upon thermal healing. This observed discrepancy could be rationalised in terms of the dynamic network differential reorganisation in comparison to conductive filler networks in the healed interfaces of our system. Here, the CNS networks necessitate precise physical contact to restore the overall transport, but the matrix moieties undergo rapid transesterification to re-establish covalent continuity across the sectioned plane. The viscoelastic flow of the matrix may cause nanosheet segregation or reorientation away from the near-fracture surface during thermal healing [60], resulting in micro-gaps (irregular continuities) that impede electron hopping. This implies that without external field support or particular alignment techniques, the topological alignment of the conductive fillers is not completely restored even when the chemical network heals. This appreciable recovery, coupled with the as-prepared composite’s ability to exhibit higher electrical conductance, enables a wide range of applications, including structural health monitoring, adaptable electronics, and components requiring regenerative electrical modules.

## 4. Conclusions

This study presents a facile approach for the fabrication of sustainable and multifunctional vitrimeric epoxy carbon nanocomposites. By introducing thiol moieties into epoxy (BADGE), the bond exchange reactions are accelerated via dynamic transesterification. We observed that the electrical and thermomechanical properties are greatly enhanced by the addition of waste-derived carbon nanosheets. Through a critical investigation of nanofiller confinement within the dynamic matrix, a hierarchical conformation of CNS has been inferred. We further observed that the graphitic structures of pyrolysis-derived CNS are highly distorted, facilitating increased interfacial effects that enhance mechanical, thermomechanical, and heat-induced effects. Correspondingly, our versatile composite exhibited a superior modulus (13.3 GPa for EC 15), which is about three times that of the pristine film, which also softens rapidly due to accelerated bond exchanges. The composites displayed excellent healing and shape recovery characteristics at a comparatively lower temperature (≥80 °C) within shorter heat treatment intervals. A superior electrical conductivity of 28 µS.m^−1^ (for EC 15), alongside exceptional flexibility and stretchability up to 276%, with partially recoverable conductance after complete healing, shows their potential for practical applications in regenerative electronic counterparts. In essence, the thermomechanical and electrical characteristics of the prepared VNCs exhibited similar characteristics to those of conventional thermosets; however, their intrinsic recoverability, endowed by DCBs, highlighted their significance. Looking forward, critical limitations, including rapid reduction in mechanical strength at near-room-temperature regimes, recovering appreciable conductance after multiple healing cycles, and further reduction in the critical temperatures, could be addressed. Therefore, this study offers a better understanding of vitrimeric carbon nanocomposite systems, which could aid in the engineering of smart, sustainable, and self-repairing structures for futuristic applications.

## Figures and Tables

**Figure 1:**
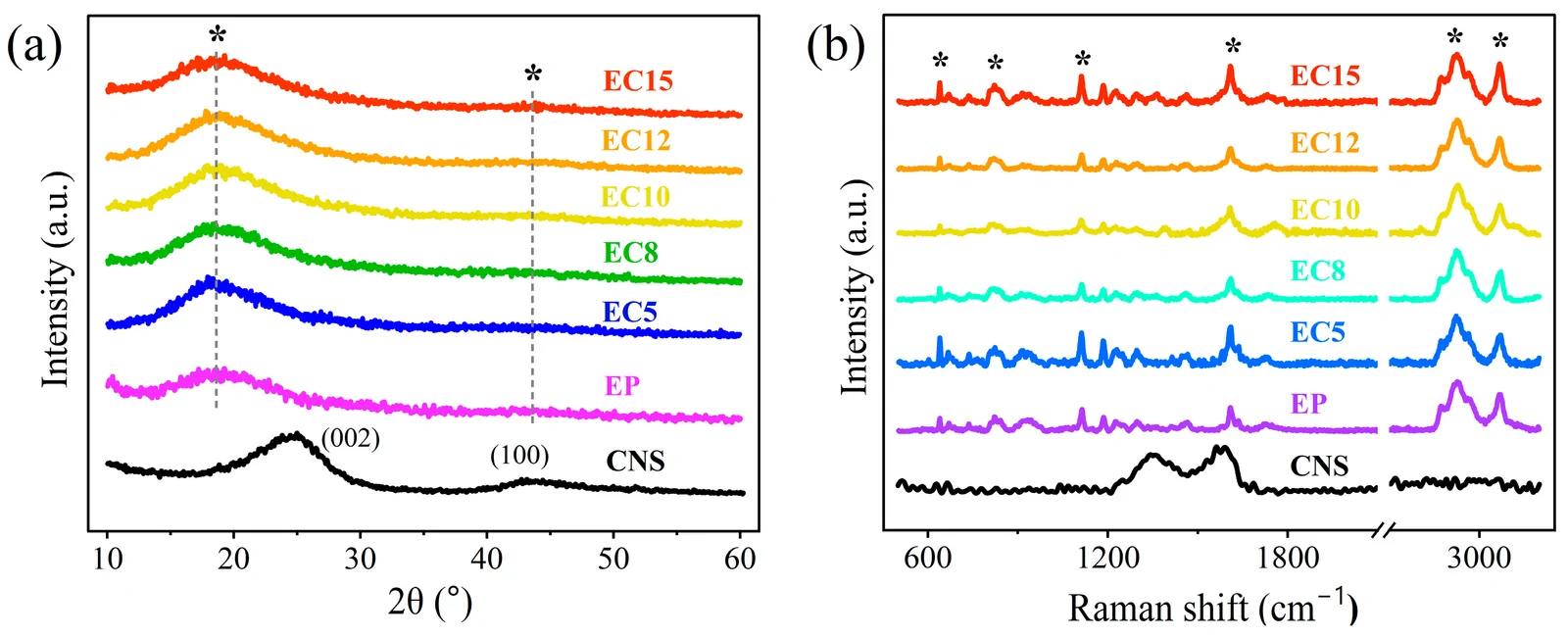
Comparative (**a**) WAXD patterns and (**b**) Raman spectra of pristine EP, CNS, and their composites. The characteristic peaks of the specimens are indicated by asterisks in the plots.

**Figure 2:**
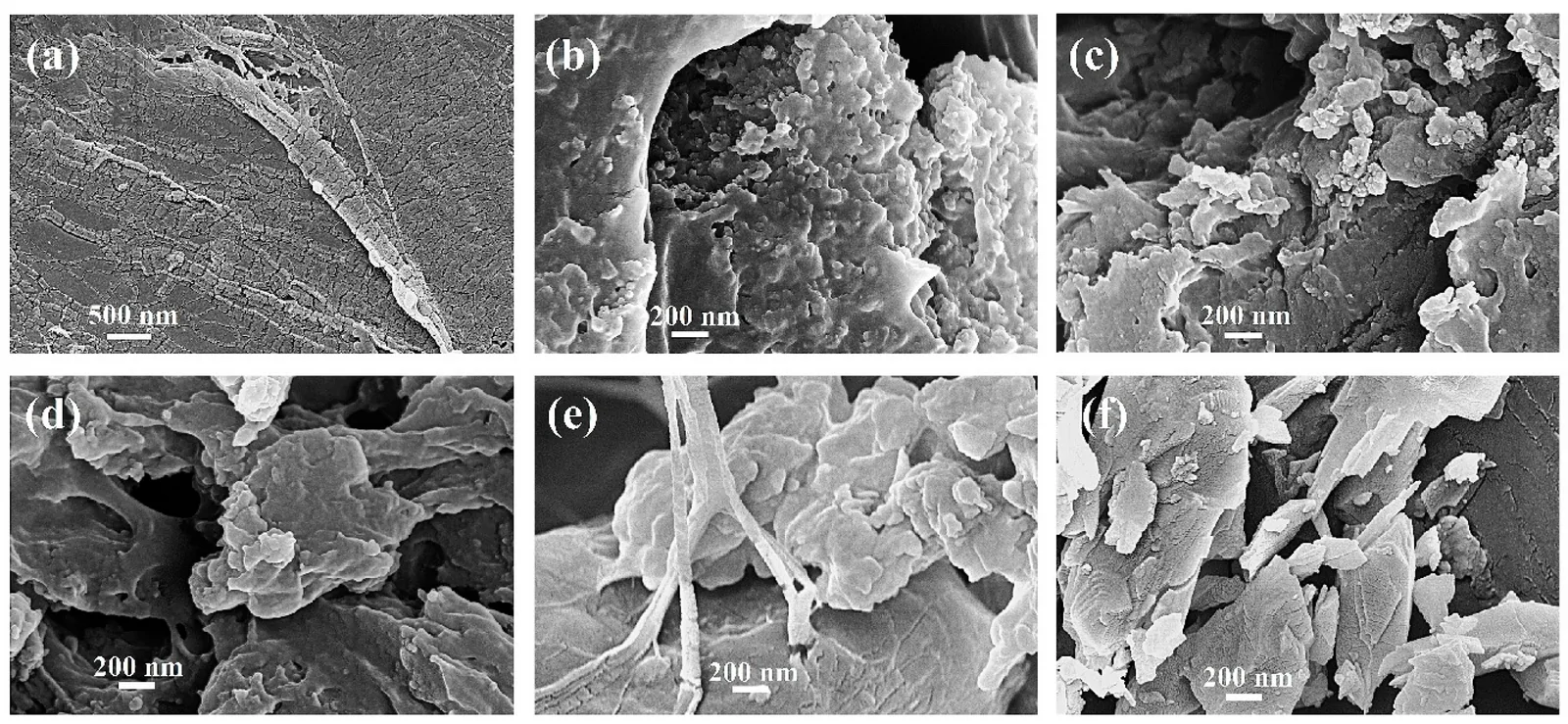
FESEM micrographs showing (**a**) surface features of the as-prepared EC5 sample. (**b**) and (**c**) show the distribution of primary filler aggregates in the EC5 film. (**d**–**f**) show secondary CNS aggregates at a higher concentration in the EC12 composite.

**Figure 3:**
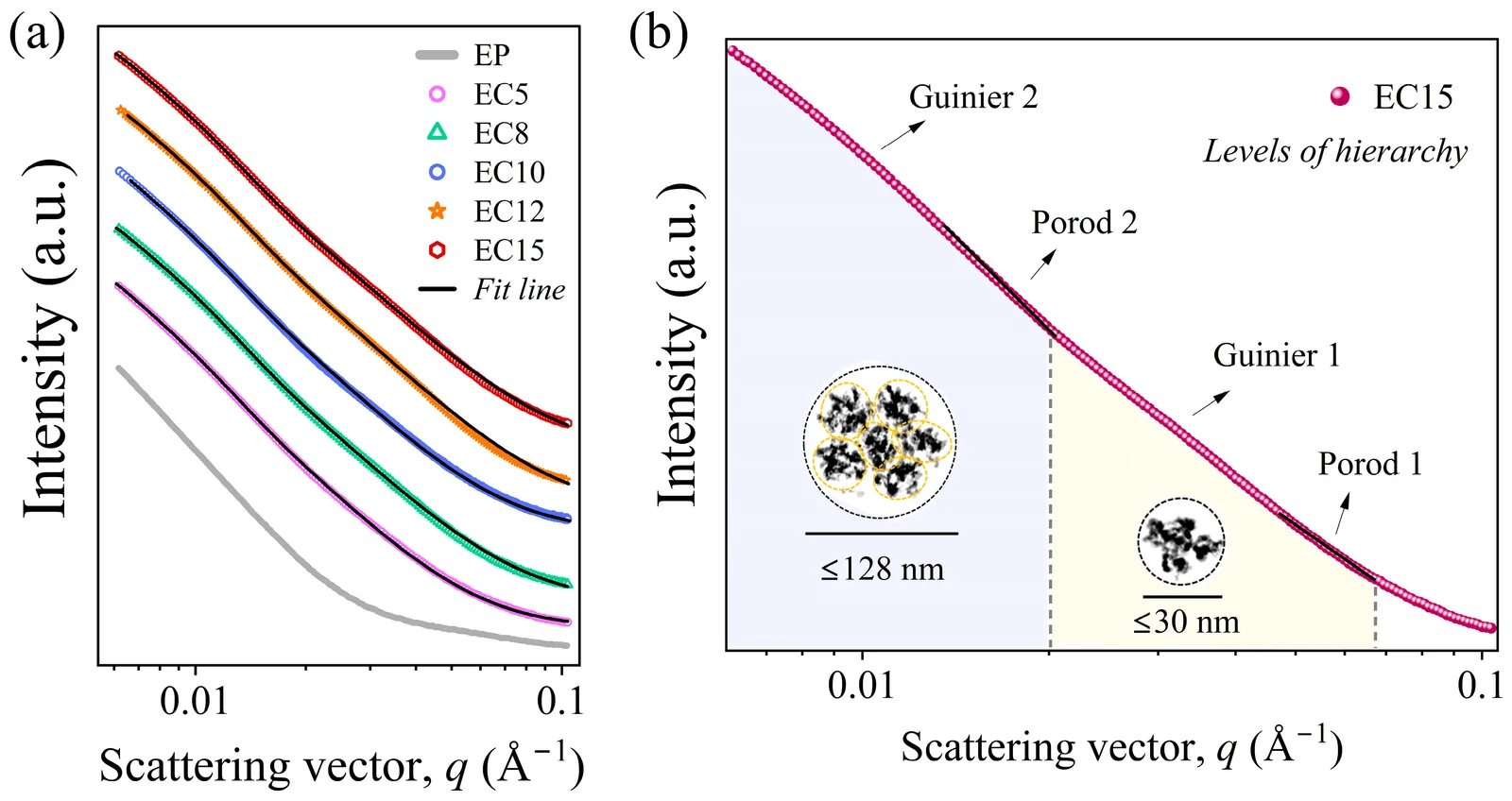
(**a**) Stacked USAXS curves for the composites represented by hollow symbols. The grey curve at the bottom indicates scattering from neat EP film. Model fits for the respective curves are indicated by solid lines. (**b**) Illustration of hierarchical levels in the unified model fit.

**Figure 4:**
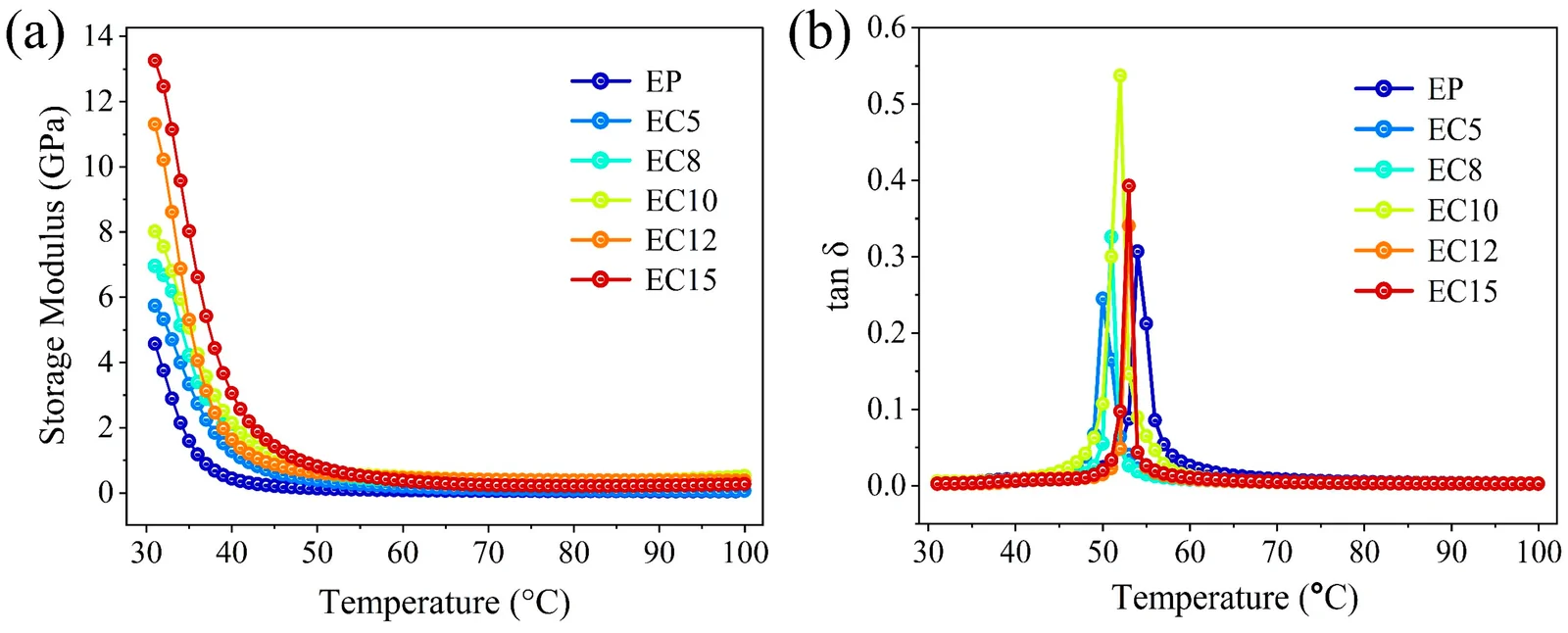
Thermomechanical characteristics of the samples: (**a**) Storage modulus and (**b**) tan δ curves, indicating filler reinforcement effects in the composites.

**Figure 5:**
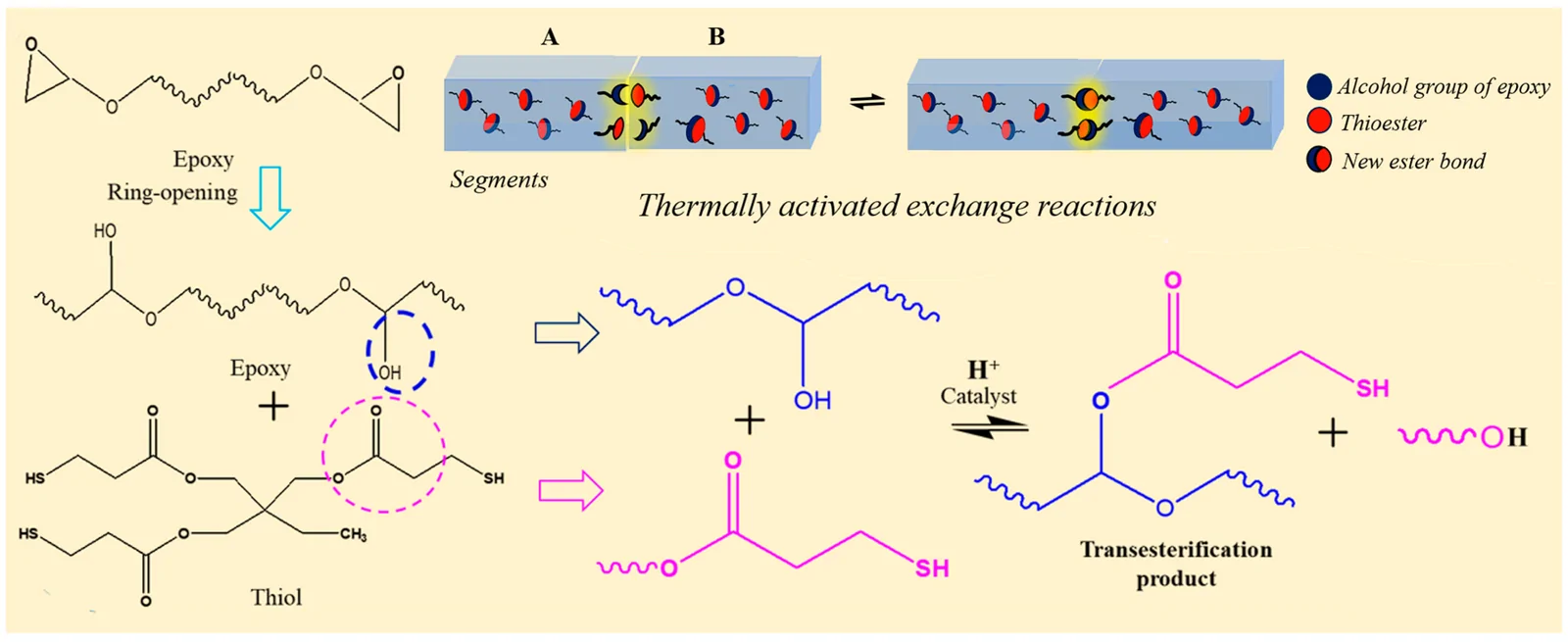
A schematic illustration of the self-healing mechanism via transesterification reaction.

**Figure 6:**
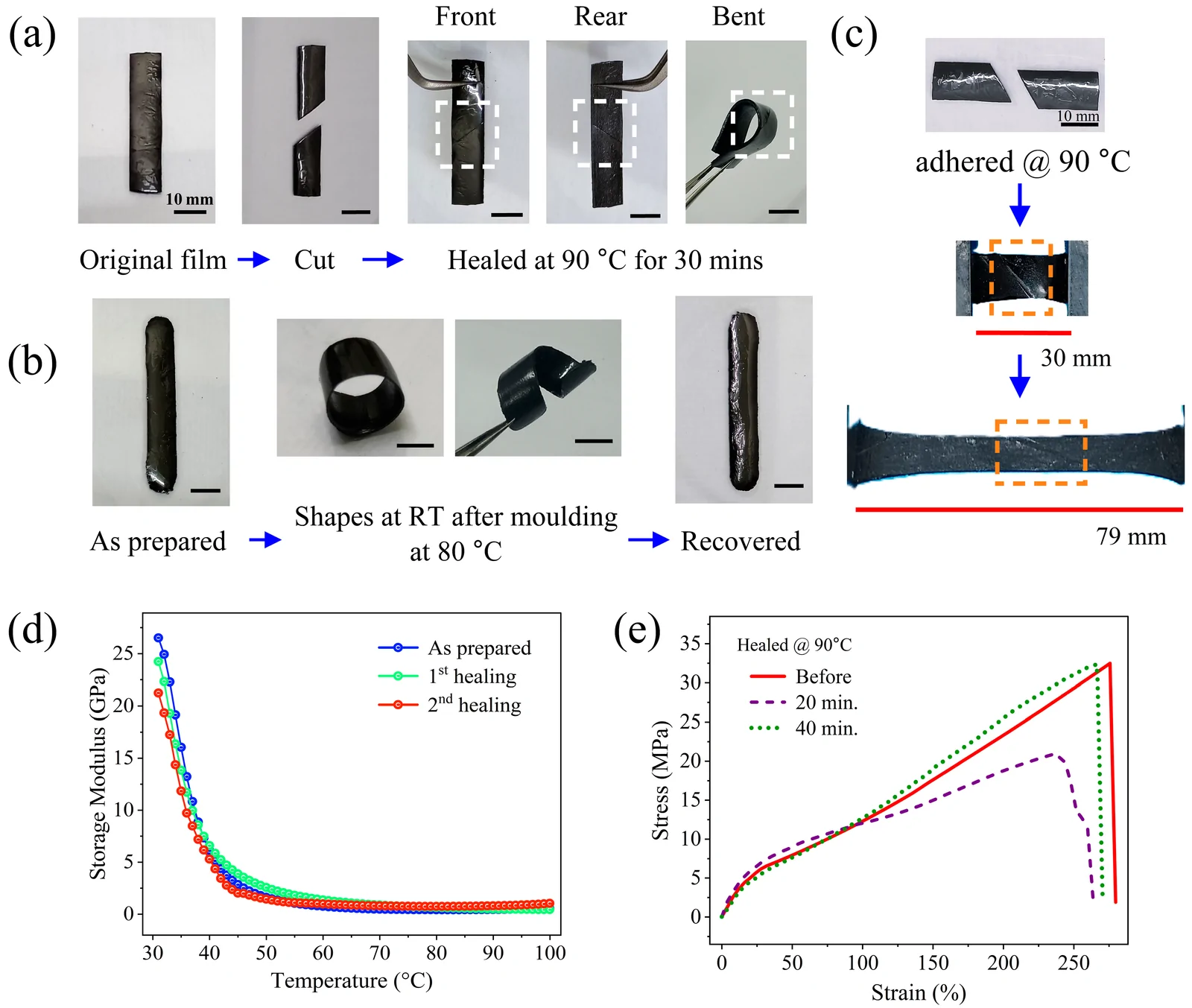
Self-healing and shape memory behaviour of EC15 nanocomposite; (**a**) Self-healing of a bisected specimen at 90 °C, with its healed site indicated by dashed boxes. (**b**) Shape manipulation tests and their recovery after heat treatment. (**c**) Surface rebonding tests of a cut sample, which, after adhering, show a 266% elongation. The adhered region is indicated by dashed boxes. (**d**) Comparison of storage modulus in the films with respect to healing cycles. (**e**) The effect of healing time on tensile characteristics indicates a nearly complete recovery of both maximum elongation (elongation at break) and tensile strength. Note: All the scale bars in black represent 10 mm.

**Figure 7:**
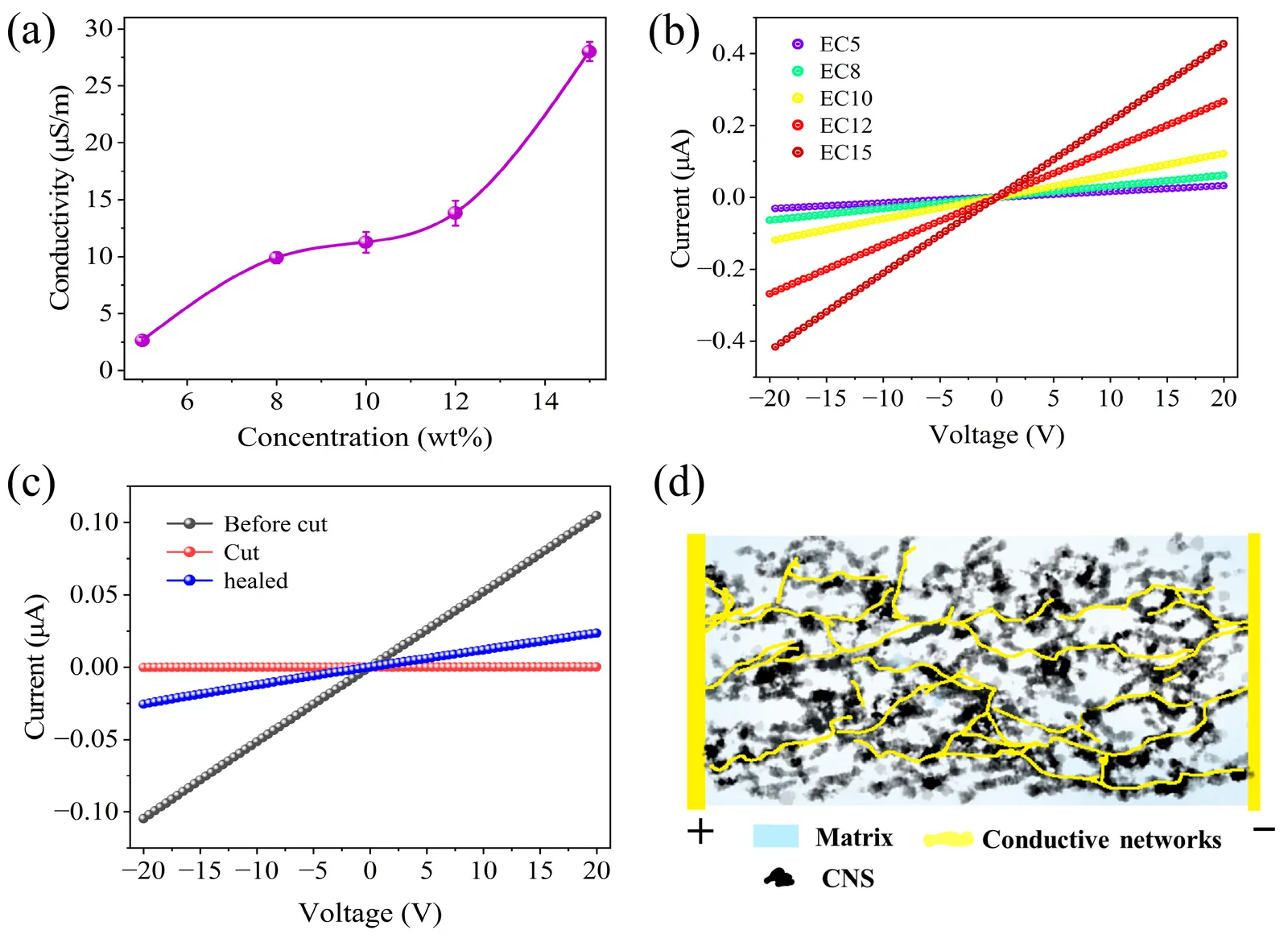
Electrical conductivity results: (**a**) Conductivity trend with filler concentration and (**b**) *V-I* curves for composites, indicating a systematic variation of slope (conductance) with concentration. (**c**) Recovery of conductivity in EC8 nanocomposite after cut-heal cycle. (**d**) A schematic illustration of percolated CNS networks facilitating electron transport.

**Table 1:** Parameters obtained from the unified model fitting.

Sample	*Rg*_1_ (Å)	*P*_1_	*Rg*_2_ (Å)	*P*_2_
EC5	241.9 ± 2.1	2.17 ± 0.24	1158.1 ± 8.1	3.09 ± 0.13
EC8	236.5 ± 1.5	2.44 ± 0.15	1202.3 ± 4.0	2.98 ± 0.29
EC10	235.5 ± 1.4	2.22 ± 0.19	1227.2 ± 1.2	3.07 ± 0.28
EC12	231.5 ± 2.4	2.53 ± 0.62	1240.8 ± 2.6	2.98 ± 0.22
EC15	237.1 ± 2.5	2.98 ± 0.32	1268.4 ± 8.5	2.93 ± 0.42

## Data Availability

The original contributions presented in this study are included in the article/Supplementary Materials. Further inquiries can be directed to the corresponding author.

## Supplementary Materials

The following supporting information can be downloaded at: https://www.mdpi.com/article/10.3390/polym18192318/s1, Figure S1: Performed shape recovery test on EC15 composite; Table S1: USAXS fitting parameters; Video S1: Shape retrieval of EC15 sample at 120 °C.
