# Supplementary material for: Study of Structural and Multifunctional Characteristics in Dynamic Transesterification-Aided Epoxy Carbon Nanocomposites
Source: Polymers (Basel). 2026 Sep 22;18(19):2318. doi: 10.3390/polym18192318 (PMC13667260; doi:10.3390/polym18192318)
Supplement: Supplementary file 1 [file polymers-18-02318-s001.zip › Supporting information_1.pdf]

SUPPORTING INFORMATION

Study of Structural and Multifunctional Characteristics in Dynamic Transesterification-Aided Epoxy Carbon Nanocomposites

Vaishnav. B[1,2], Priyanka Halsi[1], Ajay Gupta[1], Sravendra Rana[1], Sarathlal Koyiloth Vayalil[2,1]*

[1]Department of Physics, Applied Science Cluster, UPES, Dehradun, 248007, India

[2]Deutsches Elektronen-Synchrotron DESY, Notkestr. 85, 22607 Hamburg, Germany

**Corresponding author(s) email: sarathlal.koyiloth.vayalil@desy.de.*

---

1. Preparation of carbon nanofillers

The carbon nanofillers utilised in this work have been derived from discarded tyre waste via two-stage pyrolysis [1]. The acquired waste tyres were washed to remove surface-adhered debris, cut into small pieces, and oven-dried to eliminate residual moisture. The dried pieces were then combined with bentonite nanoclay, which acts as a catalyst for gradual degradation. The mixture was placed in an alumina boat and pyrolysed in a tubular furnace as follows. The material was first subjected to controlled degradation at 400 °C in a nitrogen (N_2) atmosphere for 30 minutes at a heating rate of 5 °C per minute. This facilitates the formation of a fundamental carbon structure through controlled degradation, yielding pyrochar. Later, the obtained char was rapidly heated to 900 °C at a rate of 7 °C per minute for 1 hour in an inert atmosphere. Under these conditions, the carbon skeleton undergoes layer-by-layer exfoliation, resulting in a sheet-like carbon nanostructure. The derivative was finely ground and treated with 5% nitric acid to remove pyrolytic by-products, including sulfur and other inorganic residues. After multiple washings, the desired carbon nanosheet powders were obtained.

2. Calculation of shape recovery ratio

Equation used for calculation: $R_r = \frac{\theta_m - \theta_r}{\theta_m} \times 100\ \%$

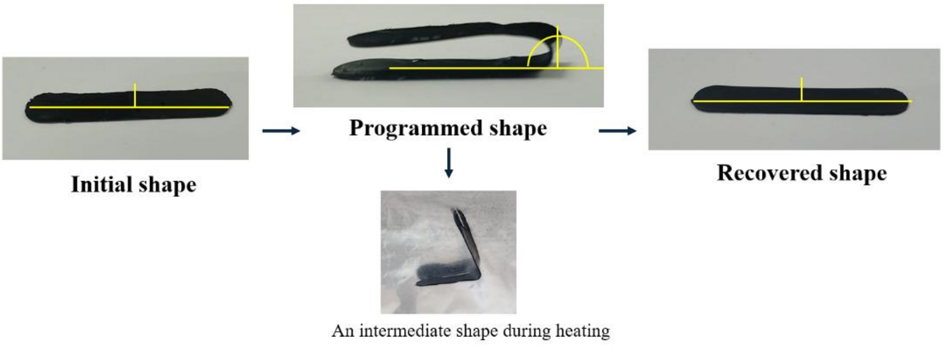

Figure S1. Performed shape recovery test on EC15 composite.

Initially deformed angle, $\theta_m = 180^o$

Angle obtained after recovering its shape by thermal activation, $\theta_r = 0^o$

$$R_r = \frac{180 - 0}{10} \times 100\ \%$$

This gives $\mathbf{R_r = 100\%}$

As shown in Figure 6(b) and the supplementary video, the deformed samples completely recover their original shape under thermal activation.

Table S1. The USAXS fitting parameters

File generated by SasView															
Chi2	Data	level	scale	rg1	rg1 (Err)	power1	power1 (Err)	B1	G1	rg2	rg2 (Err)	power2	power2 (Err)	B2	G2
7.9116	TRE5_Ang.dat	2	1	241.98	2.1035	2.1716	0.2412	-2.97E+06	2175.2	1158.1	8.1299	3.0926	0.13061	0.00093631	6.26E+09
2.0598	TRE8_Ang.dat	2	1	236.55	1.5651	2.4496	0.1471	-3.57E+06	2640.9	1202.3	4.0946	2.9837	0.289	0.0015527	1.41E+10
1.4955	TRE10_Ang.dat	2	1	235.5	1.469	2.2285	0.19602	-4.25E+08	1501.5	1227.2	1.29912	3.0723	0.2793	0.00083135	1.35E+12
7.261	TRE12_Ang.dat	2	1	231.52	2.4255	2.5341	0.61732	-1.01E+08	1285.1	1240.8	2.6981	2.9828	0.2222	0.00090267	8.07E+11
15.143	TRE15_Ang.dat	2	1	237.14	2.5689	2.9854	0.3259	-1.26E+06	896.01	1268.4	8.5031	2.9352	0.42823	0.00068913	3.24E+10

The screenshot shows the necessary output parameters generated by SasView for fitting of the composite samples.

Note: The goodness-of-fit (chi-square) values do not converge due to low error estimates. This results from the large number of data points. However, the residuals were distributed well around the fit.

References

1. Tatrari, G.; Tewari, C.; Pathak, M.; Karakoti, M.; Bohra, B.S.; Pandey, S.; SanthiBhushan, B.; Srivastava, A.; Rana, S.; Sahoo, N.G. Bulk Production of Zinc Doped Reduced Graphene Oxide from Tire Waste for Supercapacitor Application: Computation and Experimental Analysis. *J. Energy Storage* **2022**, *53*, 105098, doi: 10.1016/J.EST.2022.105098.
